# A Rare Case of Pyoderma Gangrenosum as a Complication of Ulcerative Colitis: A Case Report and Literature Review

**DOI:** 10.1002/ccr3.71291

**Published:** 2025-10-15

**Authors:** Amin Shah, Anubhav Sharma, Agnimshwor Dahal, Prinsa Shrestha, Rahul Parajuli, Rebicca Pradhan, Bibek Shrestha

**Affiliations:** ^1^ Kathmandu Medical College and Teaching Hospital Kathmandu Nepal; ^2^ Nepal Intensive Care Research Foundation Kathmandu Nepal; ^3^ Hospital for Advanced Medicine and Surgery (HAMS) Kathmandu Nepal; ^4^ Maharajgunj Medical Campus Tribhuvan University, Institute of Medicine Kathmandu Nepal

**Keywords:** inflammatory bowel disease, mesalazine, pyogenic gangrenous, ulcerative colitis

## Abstract

This case highlights pyoderma gangrenosum, a rare but serious extraintestinal manifestation of ulcerative colitis, especially in patients presenting with atypical skin ulcers and a history of inflammatory bowel disease. The pathergy phenomenon and lesions were crucial diagnostic clues, and early recognition, along with prompt initiation of corticosteroids and immunosuppressive therapy, improved outcomes.

## 1 Introduction

Ulcerative colitis (UC) is a chronic, idiopathic inflammatory bowel disease characterized by continuous mucosal inflammation extending proximally from the rectum and confined to the submucosa of the colon. This condition arises from a complex interplay of genetic, environmental, and immunological factors, resulting in a compromised colonic epithelial barrier. The annual incidence of UC ranges from 9 to 20 cases per 100,000 individuals worldwide [1]. Among patients with UC, 0.5% to 5% develop pyoderma gangrenosum (PG), a rare, severe, neutrophil‐mediated dermatological condition (Table 1). PG typically manifests on the extensor surfaces of the legs following minor trauma and begins as painful pustular lesions with poorly defined borders. Without prompt intervention, these lesions can progress to necrotic ulcers, causing significant cosmetic disfigurement, functional impairment, and permanent scarring [6, 7].

**TABLE 1:** Treatment strategies for pyoderma gangrenosum as a complication of ulcerative colitis.

Treatment class	Example(s)	Mechanism of action	Clinical use
Systemic corticosteroids	Prednisolone, Methylprednisolone	Suppress pro‐inflammatory cytokines, reduce neutrophil activity and vascular permeability	First‐line for acute PG and UC flares; provides rapid control of inflammation and promotes ulcer healing [2]
5‐ASA derivatives	Mesalazine (mesalamine)	Inhibits prostaglandin/leukotriene synthesis; activates PPAR‐γ inhibits NF‐κB	Used for mild to moderate UC; assists in mucosal healing and maintaining remission [3]
Immunosuppressants	Azathioprine	Inhibits purine synthesis; suppresses T‐lymphocyte proliferation	Steroid‐sparing agent for maintenance therapy in UC and PG [4]
Calcineurin inhibitors	Cyclosporine	Inhibits calcineurin to reduce IL‐2 and T‐cell activation	Effective in steroid‐refractory PG; rapid immunosuppression in severe cases [4, 5]
Biologic agents (Anti‐TNF‐α)	Infliximab, Adalimumab	Neutralizes TNF‐α, a key cytokine in the inflammatory pathway of PG and UC	Effective in refractory or corticosteroid‐dependent PG and UC; promotes remission
Biologic agents (Anti‐IL‐12/23)	Ustekinumab	Inhibits IL‐12 and IL‐23 to modulate innate and adaptive immune response	Considered in biologic‐experienced or TNF‐inhibitor non‐responsive patients
Topical therapy	Fusidic acid + Betamethasone valerate	Reduces local inflammation and bacterial colonization	Adjunct for localized PG lesions; enhances wound healing [4]
Anti‐inflammatory antibiotic	Dapsone	Inhibits neutrophil chemotaxis and myeloperoxidase activity	Used in mild PG or as adjunctive therapy for its antimicrobial and anti‐inflammatory properties [4]
Supportive care	Wound care, pain management, RBC transfusion	Enhances local healing, treats anemia, and prevents secondary infection	Essential for comprehensive recovery and to prevent complications such as scarring or superinfection [4]

Here, we present a rare case of a 79‐year‐old male with UC complicated by PG. This article underscores the intricate association between UC and PG, emphasizing the need for early recognition and interdisciplinary management to prevent severe complications and improve patient outcomes.

## 2 Case Presentation/Examination

A 79‐year‐old male with a known history of UC for the past 4 years presented to the outpatient department with complaints of blood in stool for 20 days and a progressively worsening wound on the anterior shin of the left leg for 15 days. The stool was liquid in consistency (Bristol Stool Chart type 7), initially occurring 8–10 times daily, later becoming uncountable, in small quantity (1–2 teaspoons each), mixed with mucus, offensive in odor, and associated with crampy lower abdominal pain. He also reported stool incontinence without associated pain during defecation.

The wound on the left anterior shin developed after trauma from a bamboo stick 15 days prior to presentation. Initially, the site showed redness and swelling, but it later progressed to form an irregular ulcer with bloody and mucopurulent discharge, foul odor, and severe pain. Despite multiple dressings at home, the wound did not subside; instead, it enlarged with undermined edges and surrounding erythema. Approximately 2 weeks after the injury, the patient developed high‐grade fever (maximum 103°F) with chills and rigors, which subsided temporarily with antipyretics. He also reported weight loss, decreased appetite, fatigue, and back pain during the same period.

**FIGURE 1:**
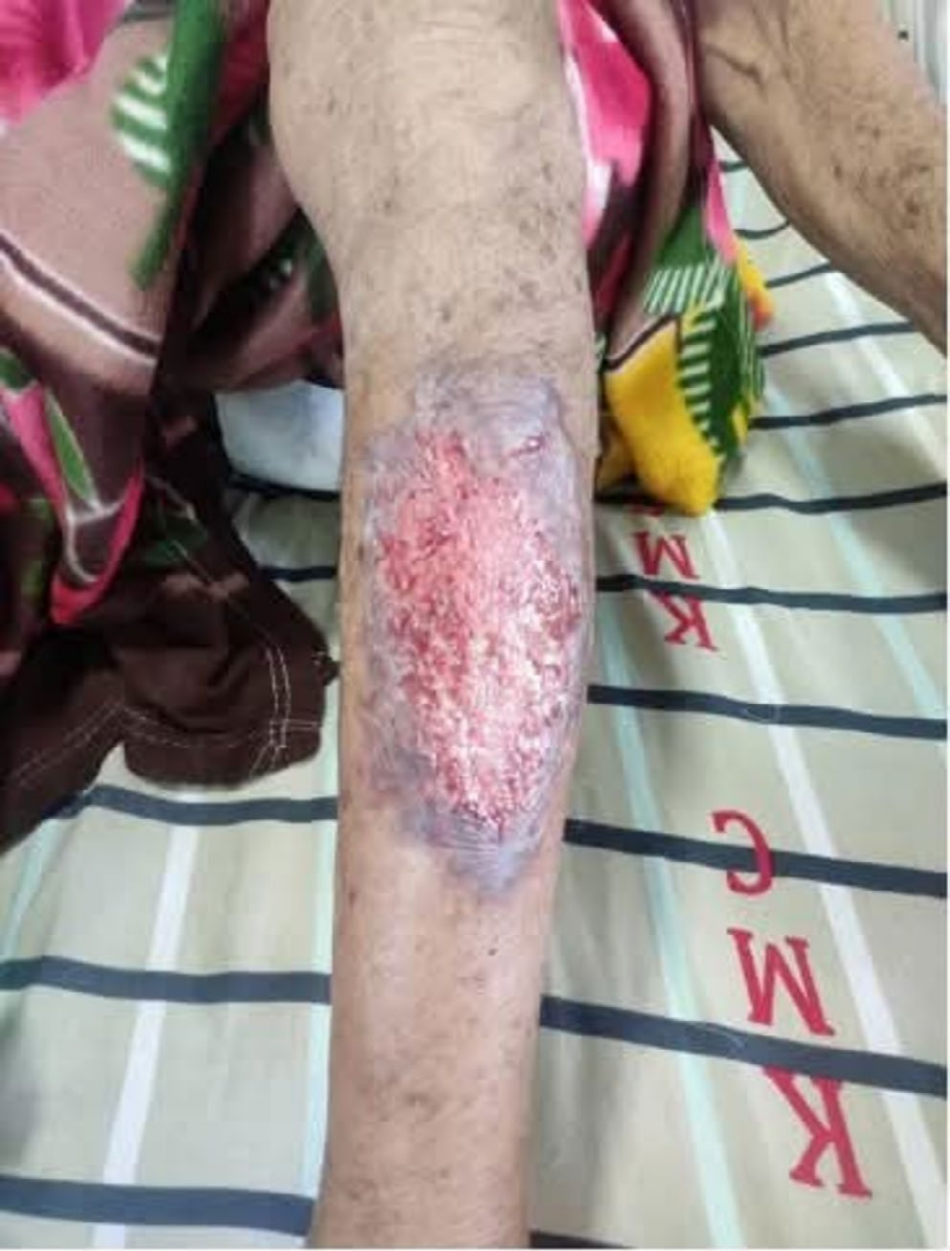
PG lesion on the left shin at presentation with a 10 × 6 cm ulcerated lesion with active oozing of blood and mucus.

During the physical examination, the patient appeared conscious, cooperative, and well‐oriented regarding time, place, and person. Pallor and localized edema were observed over the left lower leg. Moreover, a 10 × 6 cm ulcerated lesion with active oozing of blood and mucus was observed on the anterior shin of the left leg (Figure 1). The lesion had irregular edges, surrounding erythema, and an increased local temperature, suggesting inflammation. On abdominal examination, it was unremarkable, with normal bowel sounds and no tenderness. From the clinical history and examination findings, several differential diagnoses were considered for the ulcerative lesion on the patient's anterior shin. Cellulitis was initially suspected due to localized erythema, swelling, and warmth; however, the lack of systemic signs of infection such as bacteremia or positive blood cultures, along with the lesion's progression despite antibiotic treatment, ruled it out. Venous leg ulcer was another consideration, but the lesion's location on the anterior shin, its irregular borders, and the absence of clinical features of venous insufficiency, such as varicose veins or hyperpigmentation, made this unlikely. Erythema nodosum, a common dermatologic manifestation of UC, was excluded as it typically presents as tender, non‐ulcerative nodules rather than ulcerative lesions. Necrotizing fasciitis was also contemplated due to the lesion's severity, but the absence of crepitus, rapid necrosis, or gas on imaging studies negated this possibility. The presentation of an ulcer with undermined edges following minor trauma, coupled with the patient's history of UC and the pathergy phenomenon, strongly supported the diagnosis of PG.

## 3 Methods (Differential Diagnosis, Investigations and Treatment)

**FIGURE 2:**
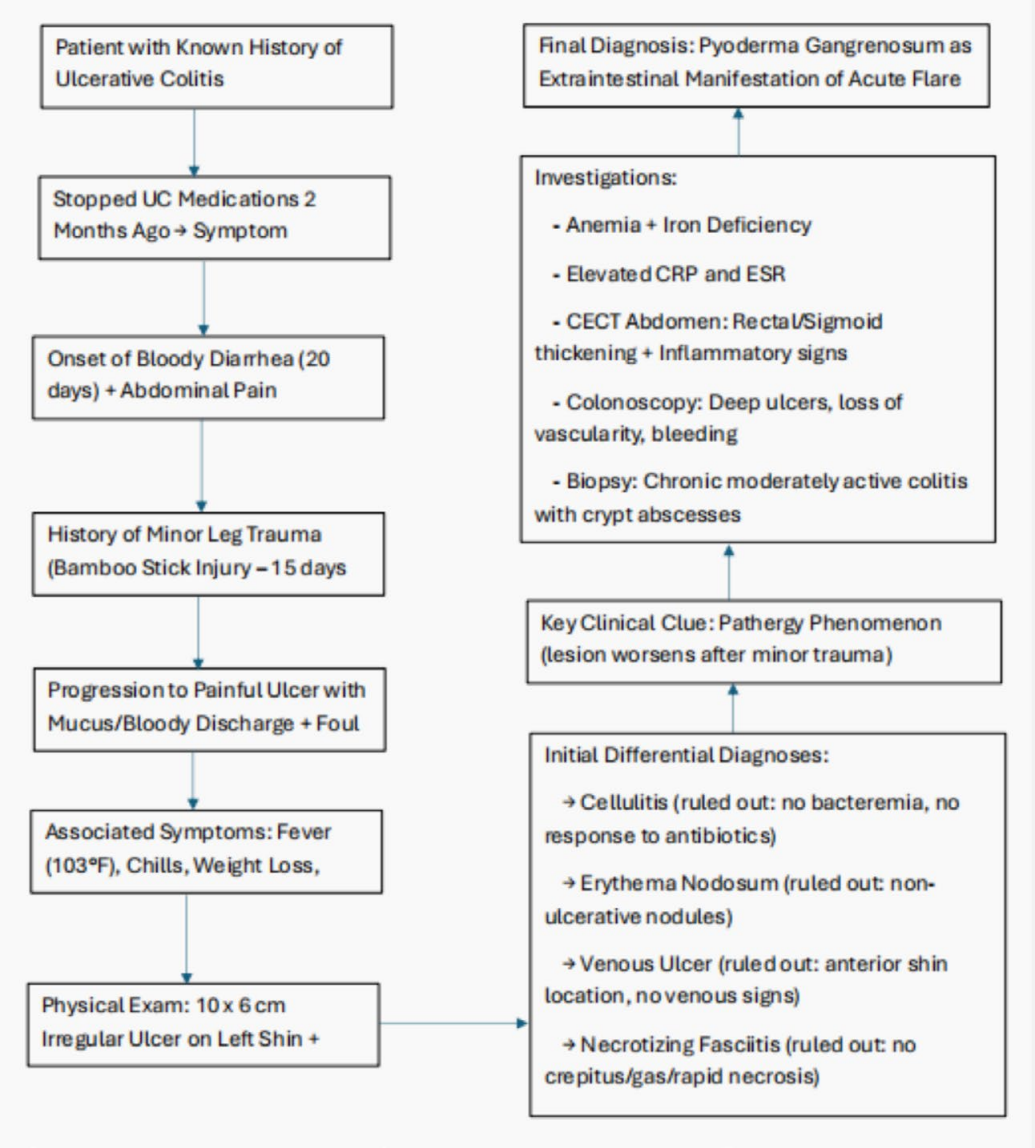
Logical progression from initial symptoms to differential diagnosis and confirmation.

**FIGURE 3:**
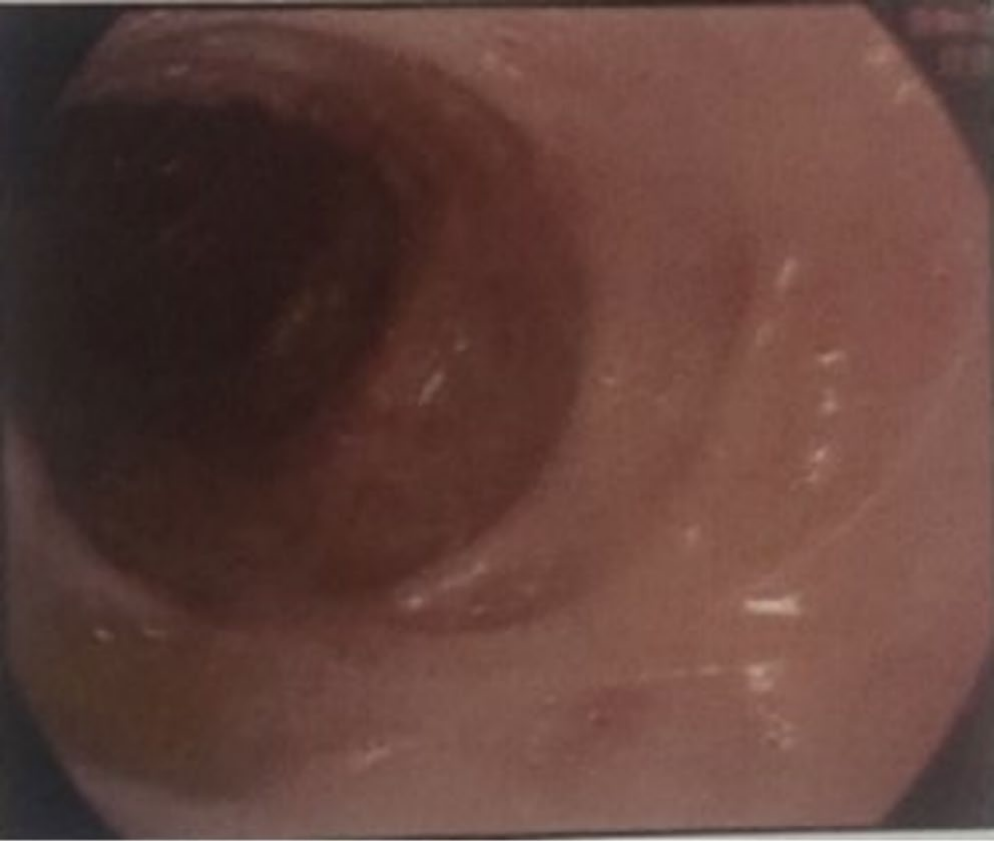
Colonoscopy showing areas of active colitis with loss of vascular pattern in the colon, consistent with findings of UC.

For further confirmation, laboratory and radiological investigations were done. Routine laboratory tests showed anemia and leukopenia, while the iron profile revealed iron deficiency with reduced ferritin and transferrin saturation, which can be attributed to significant blood loss due to frequent passage of bloody stools. Moreover, inflammatory markers, including C‐reactive protein (CRP) and erythrocyte sedimentation rate (ESR), were elevated, indicating inflammation. Logical progression from initial symptoms to differential diagnosis and confirmation has been stated in Figure 2. A contrast‐enhanced computed tomography (CECT) scan of the abdomen and pelvis revealed circumferential thickening in the rectum, sigmoid, and ascending colon, along with fat stranding and enlarged vasa recta, indicative of inflammatory bowel disease (IBD), most likely ulcerative colitis. A colonoscopy was done, which revealed a complete loss of vascular pattern, deep ulcers, luminal bleeding, and pseudo polyps in both the rectum and sigmoid colon (Figure 3). Along with the colonoscopy, a biopsy was also done, whose histopathological examination confirmed chronic moderately active colitis with crypt abscesses, architectural distortion, and basal lymphoplasmacytosis. These findings established the diagnosis of acute flare of UC. Based on clinical history, examination and laboratory findings along with the lesion's characteristic presentation at the site of minor trauma, coupled with its significant improvement following systemic corticosteroids and immunosuppressive therapy, confirmed PG as a rare extraintestinal complication of UC.

## 4 Conclusion and Results (Outcome and Follow‐Up)

**FIGURE 4:**
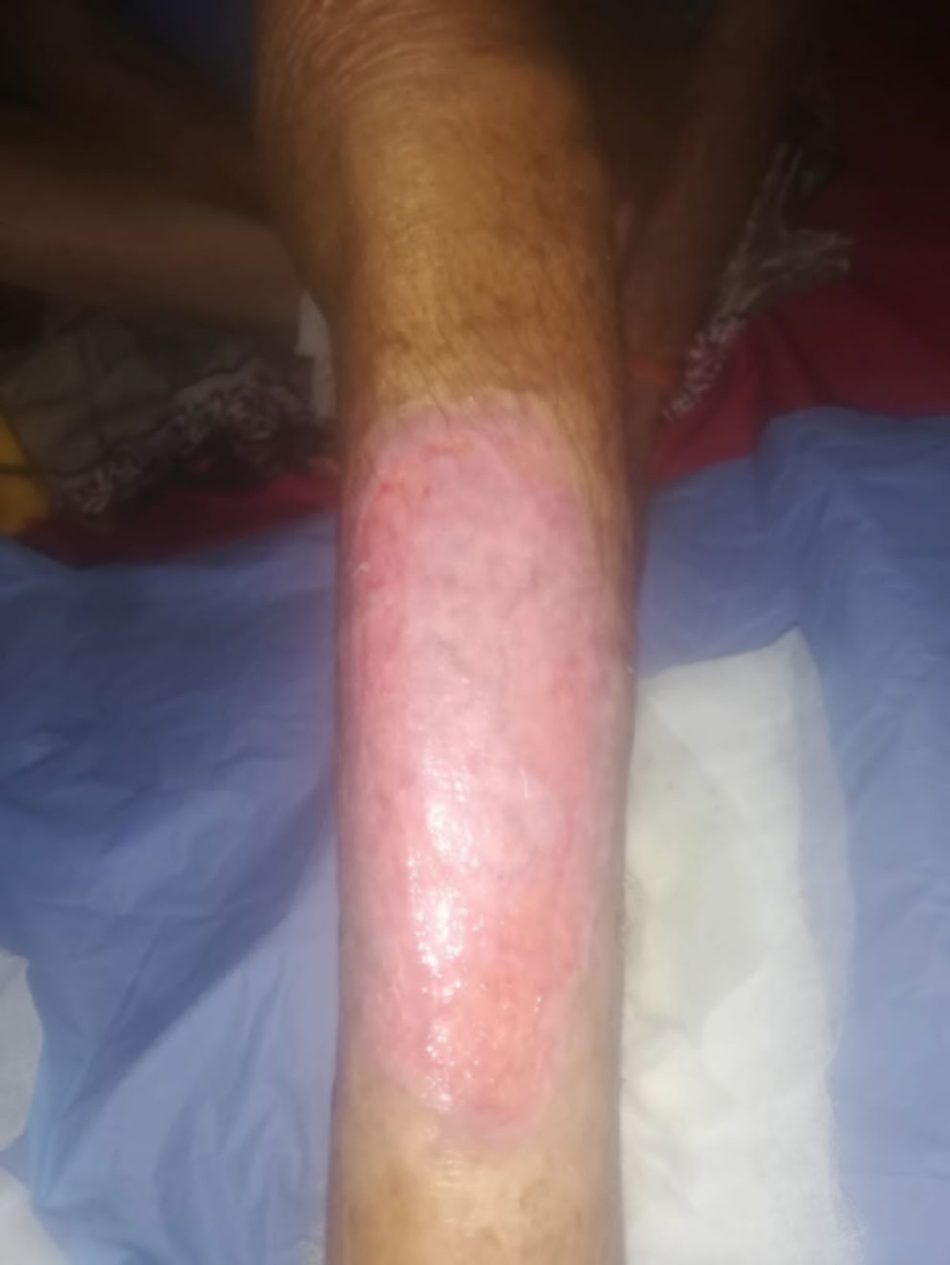
PG lesion on the left shin after treatment.

The patient was managed with Mesalazine 400 mg, Azathioprine 100 mg, and Prednisolone 50 mg for UC due to their anti‐inflammatory and immunosuppressive properties. For PG, treatment included Fusidic acid with Betamethasone valerate cream, Dapsone 100 mg, and Prednisolone 50 mg, which have anti‐inflammatory effects and promote wound healing. Following management, the wound showed a reduction in size and inflammation with visible granulation tissue, decreased erythema, and no bleeding or mucus. These findings indicated significant improvement from the presentation, and the patient was discharged after 9 days (Figure 4).

This case underscores the importance of recognizing PG as a rare but severe extraintestinal manifestation of UC. Prompt diagnosis, a multidisciplinary approach, and tailored management, including systemic corticosteroids and immunosuppressive therapies, were pivotal in achieving a favorable outcome for the patient. This case also highlights the pathergy phenomenon as a key diagnostic clue in PG and emphasizes the critical role of careful wound management in avoiding complications. By presenting this case, we aim to increase awareness among clinicians about this challenging complication, advocating for early intervention to improve patient outcomes. Further studies are warranted to better understand the pathophysiology of PG in UC and to optimize treatment strategies.

## 5 Discussion

UC is a chronic inflammatory illness characterized by recurrent and remitting inflammation of the colonic mucosa. In addition to intestinal complications, it may also manifest with extraintestinal involvement, particularly in the skin, joints, and pericardium. Among dermatological complications, PG is the second most common after erythema nodosum [8]. PG is a non‐infectious, ulcerative, inflammatory disorder often associated with systemic illnesses such as inflammatory bowel disease [9, 10]. It typically presents painful, rapidly progressing cutaneous ulcers with violaceous edges, affecting sites such as the limbs, chest, and perianal region. According to the European Crohn's and Colitis Organization, the prevalence of PG in UC patients ranges from 0.6% to 2.1%, higher than in Crohn's disease [11]. Despite its name, PG is neither infectious nor gangrenous. Its pathophysiology is multifactorial and involves abnormal neutrophilic infiltration, immune dysregulation with cytokines such as IL‐1β, IL‐8, IL‐17, and TNF‐α, as well as genetic predispositions involving pathways such as PTPN6 and PSTPIP1 [12, 13].

This case of a 79‐year‐old male with a history of UC presenting with intestinal symptoms following discontinuation of medication, along with a progressively worsening leg ulcer after minor trauma, aligns with the typical pathergy phenomenon seen in PG. The lower extremities are frequently affected, as also highlighted in the descriptive cohort study by Weizman et al. [6]. Several studies further support the correlation between UC flare‐ups and the onset of PG, which was evident in this patient who presented with severe bowel symptoms concurrent with skin lesions [6, 8, 10]. Investigations revealed anemia secondary to blood loss, colonoscopy findings of deep ulcers with pseudopolyps, and histology consistent with chronic moderately active colitis. These findings align with classical UC features described in the literature [1, 9, 10]. Ruling out infectious causes through stool culture was essential, as colonic infections may mimic UC flares [3, 14]. Based on Truelove and Witts criteria, this patient fulfilled the definition of acute severe UC due to frequent bloody stools, fever, and anemia.

Treatment of PG is challenging, particularly in the setting of UC. Corticosteroids remain the cornerstone therapy due to their potent anti‐inflammatory effect and proven efficacy in both UC and PG [2]. For refractory cases, immunosuppressive agents and biologics such as infliximab and adalimumab have demonstrated favorable outcomes [4, 5]. Cyclosporine and newer agents like ustekinumab have also been used in resistant cases [12]. In this patient, systemic corticosteroids, mesalazine, and azathioprine, along with topical and systemic agents for PG, resulted in significant improvement, eliminating the immediate need for biologics. This case emphasizes the importance of early recognition of PG as a severe extraintestinal manifestation of UC. Proper wound care, systemic therapy, and timely multidisciplinary management were key in achieving remission. Larger studies are warranted to better understand the immunopathogenesis of PG in UC, to identify predictive markers, and to optimize treatment strategies.

## Author Contributions

**Amin Shah:** conceptualization, data curation, formal analysis, methodology. **Anubhav Sharma:** investigation, supervision, validation. **Agnimshwor Dahal:** investigation, supervision, validation. **Prinsa Shrestha:** investigation, supervision, validation. **Rahul Parajuli:** investigation, supervision, validation. **Rebicca Pradhan:** investigation, supervision, validation. **Bibek Shrestha:** investigation, supervision, validation.

## Disclosure

The authors have nothing to report.

## Ethics Statement

The institutional review board (IRB) of Kathmandu Medical College and Teaching Hospital does not mandate ethical approval for case reports.

## Consent

Written informed consent was obtained from the patient for publication of this case and accompanying images, in compliance with Wiley's CCR consent form.

## Conflicts of Interest

The authors declare no conflicts of interest.

## Data Availability

The datasets used and/or analyzed during the current study are available from the corresponding author upon reasonable request.
